# Loss of TET2 impairs endothelial angiogenesis via downregulating STAT3 target genes

**DOI:** 10.1186/s13578-023-00960-5

**Published:** 2023-01-19

**Authors:** Yefei Shi, Bo Li, Xinru Huang, Wenxin Kou, Ming Zhai, Yanxi Zeng, Shuangjie You, Qing Yu, Yifan Zhao, Jianhui Zhuang, Wenhui Peng, Weixia Jian

**Affiliations:** 1 Department of Cardiology, Shanghai Tenth People’s Hospital, Tongji University School of Medicine, 301 Middle Yanchang Road, Shanghai, 200072 China; 2 Department of Endocrinology, Xinhua Hospital, Shanghai Jiaotong University School of Medicine, 1665 Kongjiang Road, Shanghai, 200092 China

**Keywords:** TET2, 5-hmC, STAT3, Angiogenesis, Ischemic diseases

## Abstract

**Background:**

Ischemic diseases represent a major global health care burden. Angiogenesis is critical in recovery of blood flow and repair of injured tissue in ischemic diseases. Ten–eleven translocation protein 2 (TET2), a member of DNA demethylases, is involved in many pathological processes. However, the role of TET2 in angiogenesis is still unrevealed.

**Methods:**

TET2 was screened out from three DNA demethylases involved in 5-hydroxylmethylcytosine (5-hmC) regulation, including TET1, TET2 and TET3. Knockdown by small interfering RNAs and overexpression by adenovirus were used to evaluate the role of TET2 on the function of endothelial cells. The blood flow recovery and density of capillary were analyzed in the endothelial cells-specific TET2-deficient mice. RNA sequencing was used to identify the TET2-mediated mechanisms under hypoxia. Co-immunoprecipitation (Co-IP), chromatin immunoprecipitation-qPCR (ChIP-qPCR) and glucosylated hydroxymethyl-sensitive-qPCR (GluMS-qPCR) were further performed to reveal the interaction of TET2 and STAT3.

**Results:**

TET2 was significantly downregulated in endothelial cells under hypoxia and led to a global decrease of 5-hmC level. TET2 knockdown aggravated the hypoxia‐induced dysfunction of endothelial cells, while TET2 overexpression alleviated the hypoxia‐induced dysfunction. Meanwhile, the deficiency of TET2 in endothelial cells impaired blood flow recovery and the density of capillary in the mouse model of hindlimb ischemia. Mechanistically, RNA sequencing indicated that the STAT3 signaling pathway was significantly inhibited by TET2 knockdown. Additionally, Co-IP, ChIP-qPCR and GluMS-qPCR further illustrated that STAT3 recruited and physically interacted with TET2 to activate STAT3 target genes. As expected, the effects of TET2 overexpression were completely suppressed by STAT3 silencing in vitro.

**Conclusions:**

Our study suggests that the deficiency of TET2 in endothelial cells impairs angiogenesis via suppression of the STAT3 signaling pathway. These findings give solid evidence for TET2 to be a therapeutic alternative for ischemic diseases.

**Graphical Abstract:**

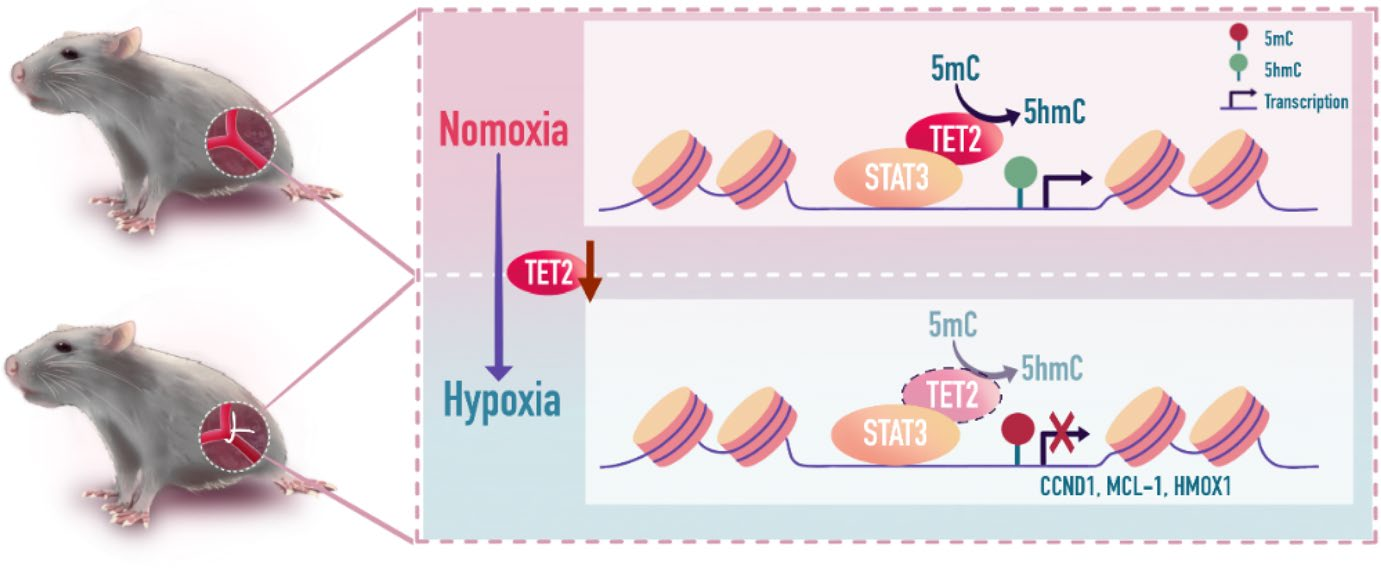

**Supplementary Information:**

The online version contains supplementary material available at 10.1186/s13578-023-00960-5.

## Background

Ischemic diseases, including coronary heart disease, peripheral artery disease and cerebrovascular disease, remain the leading causes of the global mortality and morbidity burdens [1]. Present therapeutic interventions contain dietary modifications, pharmacotherapy, and invasive surgical interventions, which can only provide symptomatic relief without addressing the injured tissue. In the past decade, proangiogenic therapy has emerged to be a promising therapeutic modality for ischemic diseases [2–4]. However, the results of preclinical and clinical trials failed to meet the expectation due to the complicated mechanisms of angiogenesis [5, 6]. These discouraging results urge the need for in-depth understanding the mechanism of angiogenesis in ischemic diseases.

Angiogenesis, the formation of new blood vessels, is a key process involved in numerous pathological conditions, such as ischemic diseases, cancer and chronic inflammatory diseases [7–9]. The molecular and cellular processes connected with angiogenesis can be divided into several steps, including endothelial cells (ECs) activation in response to angiogenic factors, basement membrane degradation via the activation of extracellular proteinase enzymes, sprouting of ECs to form a branch point in the vessel walls, and re-establishing a quiescent endothelial phenotype by recruitment of supporting pericytes and deposition of the extracellular matrix [10–12]. In these steps, ECs are the major participant in regulating the process of angiogenesis. Considering the essential role of ECs in angiogenesis, the dysfunction of ECs is closely associated with impaired angiogenic response, which features markedly in patients with ischemic diseases.

Epigenetics refers to DNA modifications can regulate gene activity without altering the DNA sequence, including DNA methylation, histone modifications and non-coding RNAs [13–15]. In these modifications, DNA methylation is the major one that has been widely investigated [13]. As for the function of DNA demethylases, Ten-eleven-translocation proteins (TET1-3) are reported to use α-ketoglutarate (α-KG) and reduced iron (Fe^2+^) as cofactors to oxidize substrates [16–19]. Following the replication-dependent dilution of the oxidized 5-methylcytosine (5-mC) or the thymine DNA glycosylase (TDG)-mediated excision of the 5-formylcytosine (5-fC) and 5-carboxylcytosine (5-caC) combined with base excision repair, TET enzymes sequentially oxidize 5-mC to 5-hmC, 5-fC, and 5-caC [20, 21]. The subsequent conversion of 5-mC to unmethylated cytosine via the base excision repair pathway results in DNA demethylation and gene activation. The 5-hmC modification and the TET enzymes have become critical activators of gene expression. Studies on TET enzymes have described that TET2 is involved in several types of cardiovascular diseases. Fuster et al. [22] reported that the deficiency of TET2 in macrophages led to an increase in NLRP3 inflammasome-mediated interleukin-1β secretion, which accelerated atherosclerosis development. In the transplant vasculopathy, Ostriker et al. [23] reported that TET2 protected against vascular smooth muscle cell apoptosis and intimal thickening. These researches showed that TET2 contributes significantly to cardiovascular disease. Recent studies demonstrated that TET2 mediated the alterations of 5-hmC in response to glucose in ECs, as TET1 and TET3 were barely detectable in ECs [24], and TET2 could be regulated by hypoxia in some cancer cell lines [25]. Therefore, we hypothesized that ischemia-related pathologic stimuli may regulate the production of 5-hmC, and TET2 may be involved in mediating the function of ECs in ischemic diseases.

In this study, we systematically investigated the role of TET2 in ECs under hypoxia. By knockdown or overexpression of TET2, we uncovered that TET2 could regulate the hypoxia-induced dysfunction of ECs. Additionally, we reported that TET2 was recruited by STAT3 to modulate the target genes via DNA demethylation. Overall, this study provided deeper insights into the regulation of 5-hmC level in ECs function and presented a new effective strategy that the TET2-5-hmC pathway may be a potential treatment for ischemic disease.

## Results

### Hypoxia impairs angiogenesis and downregulates TET2 expression in ECs

**Fig. 1:**
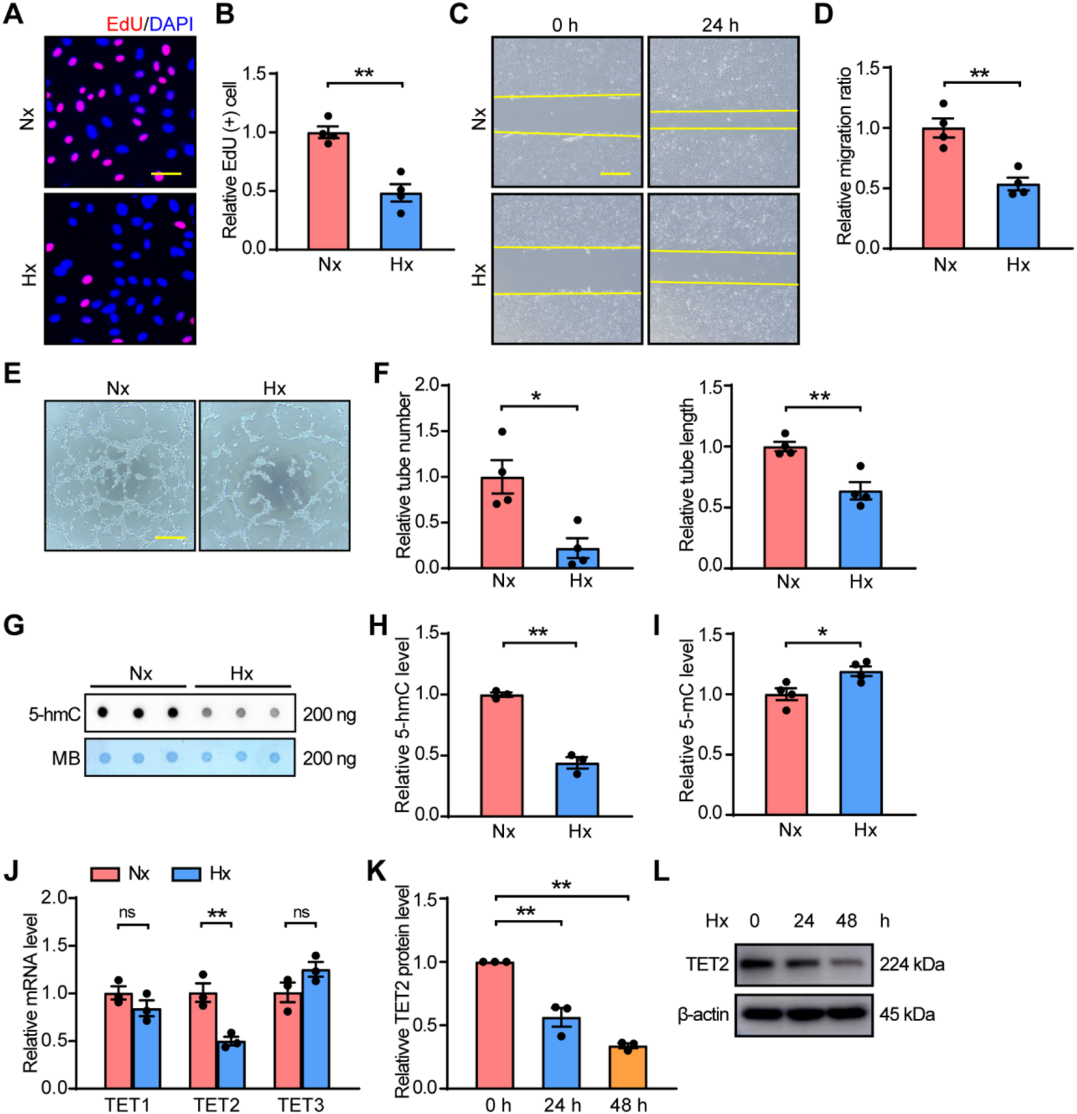
Hypoxia impairs angiogenesis and downregulates TET2 expression in HUVECs. **A**, **B** EdU assay, **C**, **D** Scratch assay and **E**, **F** Tube formation assay of HUVECs under normoxia and hypoxia. **G**, **H** Dot blot assay of global 5-hmC and **I** ELISA assay of global 5-mC using genomic DNA from HUVECs treated with normoxia and hypoxia. **J** Quantification of the mRNA expression levels of TET1, TET2 and TET3 under normoxia and hypoxia. **K**, **L** Western blot of TET2 expression levels under hypoxia for 0 h, 24 h and 48 h. n = 3–4 per group. **B**, **D**, **F**, **H**, **I**, **J** Unpaired Student’s *t*‐test. **K** One-way ANOVA with Bonferroni post hoc test. *ns* no significant; *, *P* < 0.05; **, *P* < 0.01; *Nx* normoxia, *Hx* hypoxia. **A** Scale bar, 50 μm. **C**, **E** Scale bar, 100 μm

To determine the role of 5-hmC in angiogenesis, we investigated how hypoxia affected the angiogenic phenotypes of human umbilical vein endothelial cells (HUVECs), including cell proliferation, migration, and tube formation. We first measured the expression of HIF-1α and the changes of VEGF, GLUT1, PDK1 to confirm that the hypoxia model was successfully performed with HUVECs in vitro (Additional file 1: Fig. S1A–C). Cell proliferation was evaluated by EdU assay, and the result showed that the ratio of proliferating cells was remarkably decreased under hypoxia (Fig. 1A, B). The ability of migration was detected by scratch assay and hypoxia significantly reduced the area of wound healing (Fig. 1C, D). Furthermore, the capacity of tube formation was examined and the data suggested that the tube number and tube length were notably impaired by hypoxia (Fig. 1E, F). In sum, these data show that hypoxia inhibits the ability of angiogenesis in HUVECs.

To determine whether 5-hmC was changed in HUVECs under hypoxia, we carried out dot blot assay to evaluate the level of 5-hmC, and the results revealed that 5-hmC abundance was significantly reduced under hypoxia (Fig. 1G, H). However, the global 5-mC was slightly increased in ELISA assay (Fig. 1I), presumably because 5-mC was more abundant than 5-hmC [28]. As 5-hmC was regulated by TET enzymes, we performed RT-qPCR to evaluate the expression levels of TET enzymes (TET1-3) in HUVECs under hypoxia. The data showed that TET2 was significantly downregulated under hypoxia, while the expression of TET1 and TET3 had no significant change (Fig. 1J), suggesting that the variation of global 5-hmC was mainly due to the alteration of TET2. Furthermore, the results of western blot (Fig. 1K, L) showed that TET2 displayed a time-dependent decreased in HUVECs under hypoxia. Taken together, these data suggest that TET2 is closely associated with angiogenesis in ischemic diseases.

### TET2 regulates the hypoxia‐induced dysfunction of ECs

**Fig. 2:**
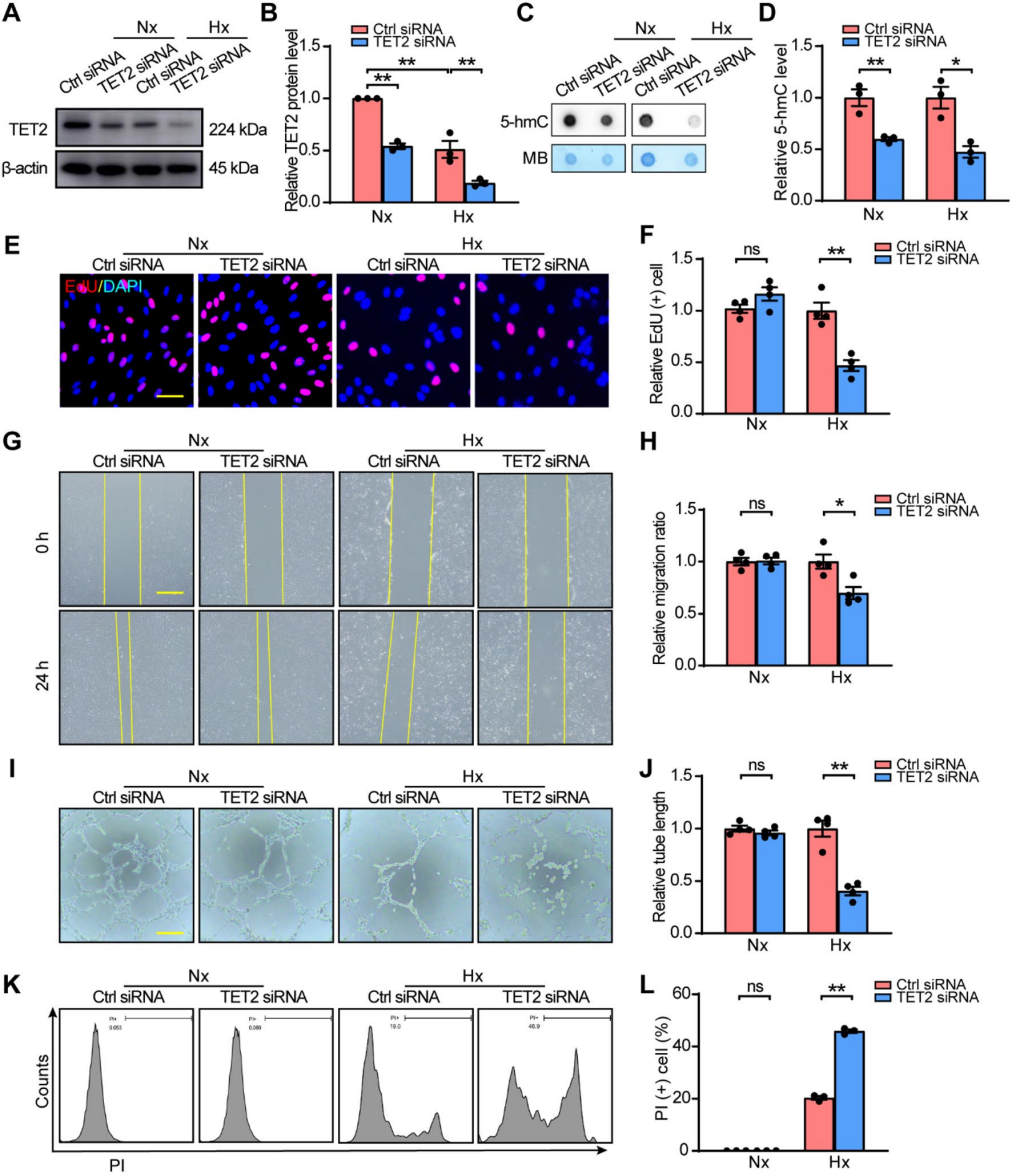
TET2 knockdown aggravates the hypoxia‐induced dysfunction of HUVECs. **A**, **B** Western blot of HUVECs transfected with Ctrl siRNA or TET2 siRNA under normoxia and hypoxia. **C**, **D** Dot blot assay of global 5-hmC using genomic DNA from HUVECs transfected with Ctrl siRNA or TET2 siRNA under normoxia and hypoxia. **E**, **F** EdU assay, **G**, **H** Scratch assay, **I**, **J** Tube formation assay, and **K**, **L** Apoptosis assay of HUVECs transfected with Ctrl siRNA or TET2 siRNA under normoxia and hypoxia. n = 3–4 per group. **B** Two-way ANOVA with Bonferroni post hoc test. **D**, **F**, **H**, **J**, **L** Unpaired Student’s *t*‐test. *ns* no significant; *, *P* < 0.05; **, *P* < 0.01; *Nx* normoxia, *Hx* hypoxia. **E** Scale bar, 50 μm. **G**, **I** Scale bar, 100 μm

To figure out the possible role of TET2 in HUVECs, TET2 was knocked down using small interfering RNAs (siRNAs). Western blot was performed to confirm that TET2 was successfully downregulated under normoxia and hypoxia (Fig. 2A, B). Next, the global 5-hmC level of HUVECs under normoxia and hypoxia was examined after transfection with siRNAs. Dot blot assay revealed reduced 5-hmC abundance after TET2 knockdown (Fig. 2C, D). Furthermore, the effects of TET2 knockdown on angiogenesis were examined in vitro. The EdU assay showed that TET2 knockdown impaired the proliferation of HUVECs under hypoxia but not normoxia (Fig. 2E, F). Similarly, silencing TET2 attenuated HUVECs’ capacities of migration (Fig. 2G, H) and tube formation (Fig. 2I, J) under hypoxia but not normoxia. Moreover, apoptosis was analyzed by flow cytometry (gating strategy presented in Additional file 1: Fig. S2) and the result showed that knocking down TET2 enhanced the apoptosis of HUVECs under hypoxia but not normoxia (Fig. 2K, L).

To further understand the role of TET2 in angiogenesis, adenoviruses were used to mediate TET2 overexpression. The results of western blot (Additional file 1: Fig. S3A, B) showed that TET2 was successfully overexpressed in HUVECs. In addition, the dot blot assay illustrated that the global 5-hmC level was increased by the overexpression of TET2 under normoxia and hypoxia (Additional file 1: Fig. S3C, D). Next, the EdU assay, scratch assay, tube formation assay and apoptosis assay were used to evaluate the effects of TET2 overexpression in HUVECs. The EdU assay indicated that TET2 overexpression improved HUVECs proliferation (Additional file 1: Fig. S3E, F) under hypoxic condition but not normoxia. Moreover, overexpression of TET2 promoted the capacities of migration (Additional file 1: Fig. S3G, H) and tube formation (Additional file 1: Fig. S3I, J) of HUVECs under hypoxia but not normoxia. In addition, increased TET2 alleviated hypoxia-induced apoptosis in HUVECs but not normoxia (Additional file 1: Fig. S3K, L). These data collectively indicate that TET2 is critical in hypoxia‐induced dysfunction of endothelial angiogenesis.

### Deficiency of TET2 impairs pathological angiogenesis

**Fig. 3:**
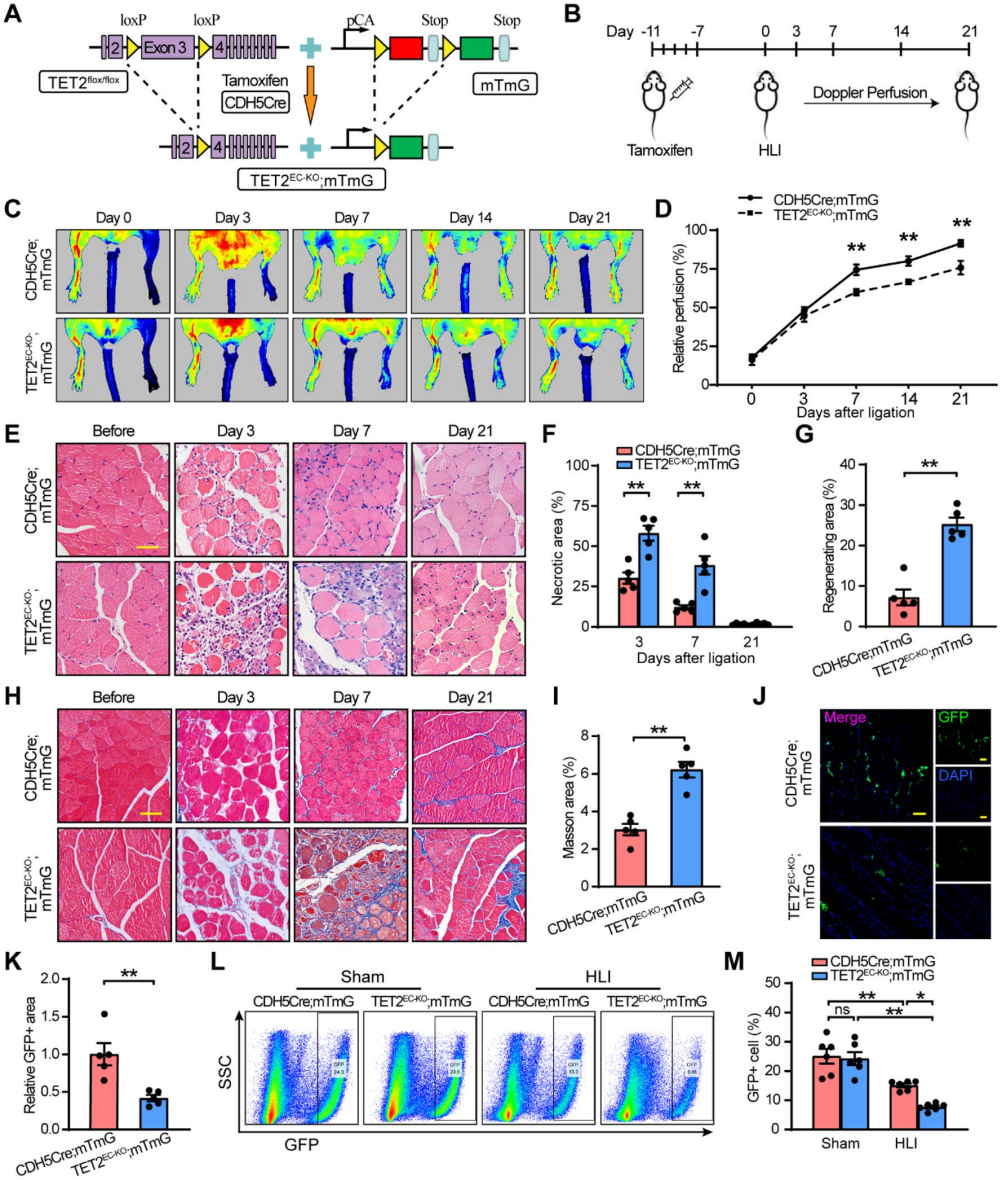
Deficiency of TET2 impairs blood flow recovery and density of capillary in the HLI model. **A** Genomic structure of TET2^EC−KO^ mice. **B** The time points of treatments in mice. **C**, **D** The blood flow recovery of CDH5Cre; mTmG and TET2^EC−KO^; mTmG mice. **E**, **F**, **G** Representative hematoxylin–eosin (H & E) staining images and quantification of the necrotic area at the indicated times, as well as regenerating area 21 days after HLI. **H**, **I** Representative masson staining images at the indicated times and quantification of regenerating area 21 days after HLI. **J**, **K** Representative staining images of GFP-positive cells in the cross section of gastrocnemius muscles of the ligated hindlimb 14 days after HLI. GFP (green), DAPI (blue). **L**, **M** Flow cytometry analysis of GFP-positive cells in the gastrocnemius muscles (14 days). n = 5–7 mice per group. **D**, **F**, **G**, **I**, **K** Unpaired Student’s *t*‐test. **M** Two-way ANOVA with Bonferroni post hoc test. *ns* no significant; *, *P* < 0.05; **, *P* < 0.01; *HLI* hindlimb ischemia, **E,** **H** Scale bar, 20 μm. **J** Scar bar, 100 µm

To investigate the role of TET2 in post‐ischemic angiogenesis in the hindlimb ischemia (HLI) model and better trace endothelial cells, endothelial cells specific TET2-deficient mice (TET2^EC−KO^; mTmG) were constructed, of which endothelial cells were labeled with GFP after being induced with tamoxifen (Fig. 3A). PCR was used to detect the alleles from mice tail and the successful targeting was verified at DNA (Additional file 1: Fig. S4A). After being induced by tamoxifen for 5 consecutive days, the endothelial cells of gastrocnemius muscle were successfully labelled with GFP (Additional file 1: Fig. S4B), which was more obvious in the intima of the aorta (Additional file 1: Fig. S4C). Furthermore, GFP-positive cells of gastrocnemius muscle were separated by flow cytometry and the qPCR result of the separated cells showed that TET2 level was reduced by more than 50% in TET2^EC−KO^; mTmG mice (Additional file 1: Fig. S4D). One week after injection with tamoxifen, HLI model was conducted and the blood flow rate was evaluated by a laser Doppler scanning system over the duration of 21 days after HLI (Fig. 3B). On 7–21 days after HLI, TET2^EC−KO^; mTmG mice exhibited significantly decreased blood flow recovery rates compared with control mice (Fig. 3C, D). We also observed more severe myocyte necrosis 3 days and 7 days after HLI (Fig. 3E, F), and larger area of intense regeneration 21 days after HLI in TET2^EC−KO^; mTmG mice (Fig. 3E, G). Meanwhile, a greater degree of fibrosis was observed in TET2^EC−KO^; mTmG mice 21 days after HLI (Fig. 3H, I). Moreover, the GFP-positive ratio, which indicated the densities of capillaries and small arteries, was also significantly downregulated in the TET2^EC−KO^; mTmG mice (Fig. 3J, K). To better assess the ratio of endothelial cells, flow cytometry was conducted to trace GFP-positive cells in the hindlimb (gating strategy presented in Additional file 1: Fig. S4E). The flow cytometry analysis demonstrated that TET2^EC−KO^; mTmG mice had fewer GFP-positive cells compared with control mice (Fig. 3L, M).

As angiogenesis was critical for tumor growth, allograft transplantation of tumor was often used as a model to mimic a complicated microenvironment [29, 30]. Here we investigated the function of TET2 in tumor angiogenesis using melanoma cells (B16F10). Indeed, tumors in TET2^EC−KO^ mice showed significantly decreased final tumor size and tumor mass as compared with TET2^flox/flox^ mice (Additional file 1: Fig. S5A, B). Simultaneously, tumors in TET2^EC−KO^ mice grew slower than the TET2^flox/flox^ mice as quantified by tumor size (Additional file 1: Fig. S5C). In addition, the result of the staining intensity for the endothelial marker CD31 showed that there was less vessel density in TET2^EC−KO^ mice than in the control mice (Additional file 1: Fig. S5D, E). These data of the two models illustrate that the deficiency of TET2 impairs pathological angiogenesis.

### TET2 regulates STAT3 target genes in ECs

**Fig. 4:**
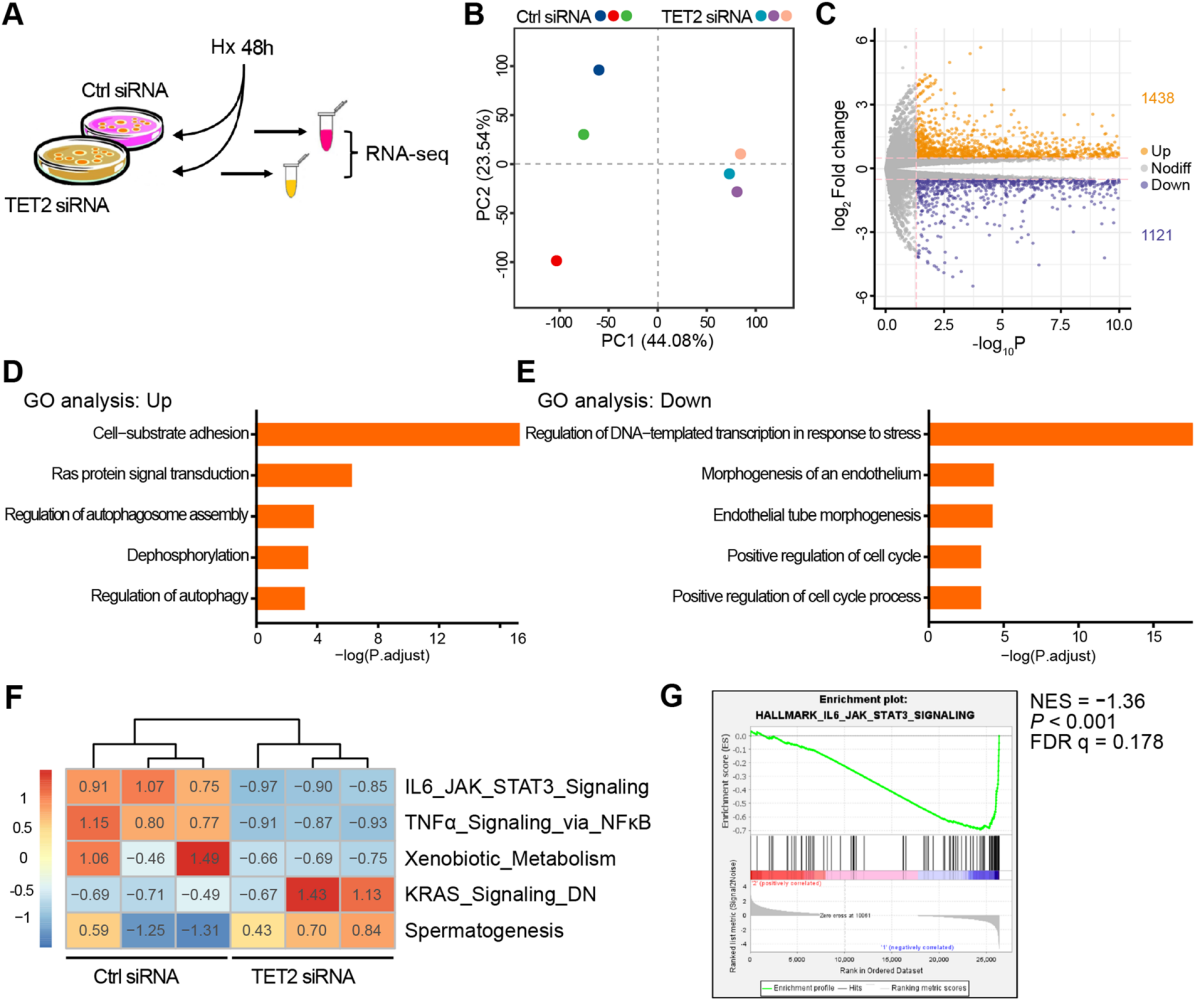
TET2 regulates STAT3 target genes in ECs. **A** Schematic illustration of samples preparation for RNA sequencing. **B** Principal component analysis for HUVECs transfected with Ctrl siRNA or TET2 siRNA. **C** Volcano plots to determine differentially expressed genes for HUVECs transfected with Ctrl siRNA or TET2 siRNA. GO enrichment analysis of the upregulated genes **D** and downregulated genes **E** in different biologic response of AdTET2 compared with AdCtrl. **F** GSVA of the expression of several genes were performed with role in different signaling pathways from RNA-sequencing. **G** GSEA of the genes in the IL-6_JAK_STAT3 pathway generated from RNA sequencing

To investigate the mechanism of TET2 in ECs under hypoxia, RNA sequencing was conducted in HUVECs with TET2 knockdown to explore the potential mechanism (Fig. 4A). The principal component analysis revealed clear differences between the two groups, the TET2 siRNA group clustered from the control group, suggesting transcriptional heterogeneity (Fig. 4B). To explore genes that were affected by TET2 knockdown, we performed differentially mRNA expression analysis in the TET2 siRNA group, comparing with ctrl siRNA group. The results showed that 1438 genes were upregulated and 1121 genes were downregulated (Fig. 4C). In addition, gene ontology (GO) analysis was conducted to analyze the upregulated and downregulated genes by TET2 knockdown respectively. GO analysis showed that upregulated genes primarily affected adhesion, Ras, autophagy, and dephosphorylation (Fig. 4D), while downregulated genes primarily affected transcription, endothelium and cell cycle (Fig. 4E). We also performed GSVA of differentially expressed gene enrichment. The result showed that STAT3 pathway, TNFα pathway and metabolism were downregulated with the knockdown of TET2, while KRAS pathway and spermatogenesis were partly upregulated (Fig. 4F). Of note, the STAT3 singling pathway, a key regulator of angiogenesis [31], was significantly suppressed in GSVA. To further identify the alteration of the STAT3 signaling pathway, Gene Set Enrichment Analysis (GSEA) was performed to assess the change of this pathway. As expected, the STAT3 signaling pathway was remarkably suppressed by TET2 knockdown in GSEA (Fig. 4G). Obtained results suggest that STAT3 singling pathway may be the potential target of TET2.

### TET2 is recruited by STAT3 and activates its target genes

As a broad epigenetic modifier, we hypothesized that TET2 could directly modulate the expression of STAT3. The result of western blot illustrated that the deficiency of TET2 did not affect either the expression or the phosphorylation level of STAT3 (Additional file 1: Fig. S6). Our previous studies identified that NLRC5 could affect the translocation of STAT3 [32], so we examined whether TET2 had a similar function to NLRC5. To verify this hypothesis, the expression of TET2 in HUVECs was knocked down by siRNAs and HUVECs were stimulated with interleukin-6 (IL-6). The extraction of cytoplasmic and nuclear proteins assay showed that STAT3 stared to translocate to the nucleus after 15 min and peaked at 30 min. However, there was no difference between the group transfected with TET2 siRNA and control group (Additional file 1: Fig. S7A–C). Immunofluorescence also showed that there was no difference between the two groups (Additional file 1: Fig. S7D).

**Fig. 5:**
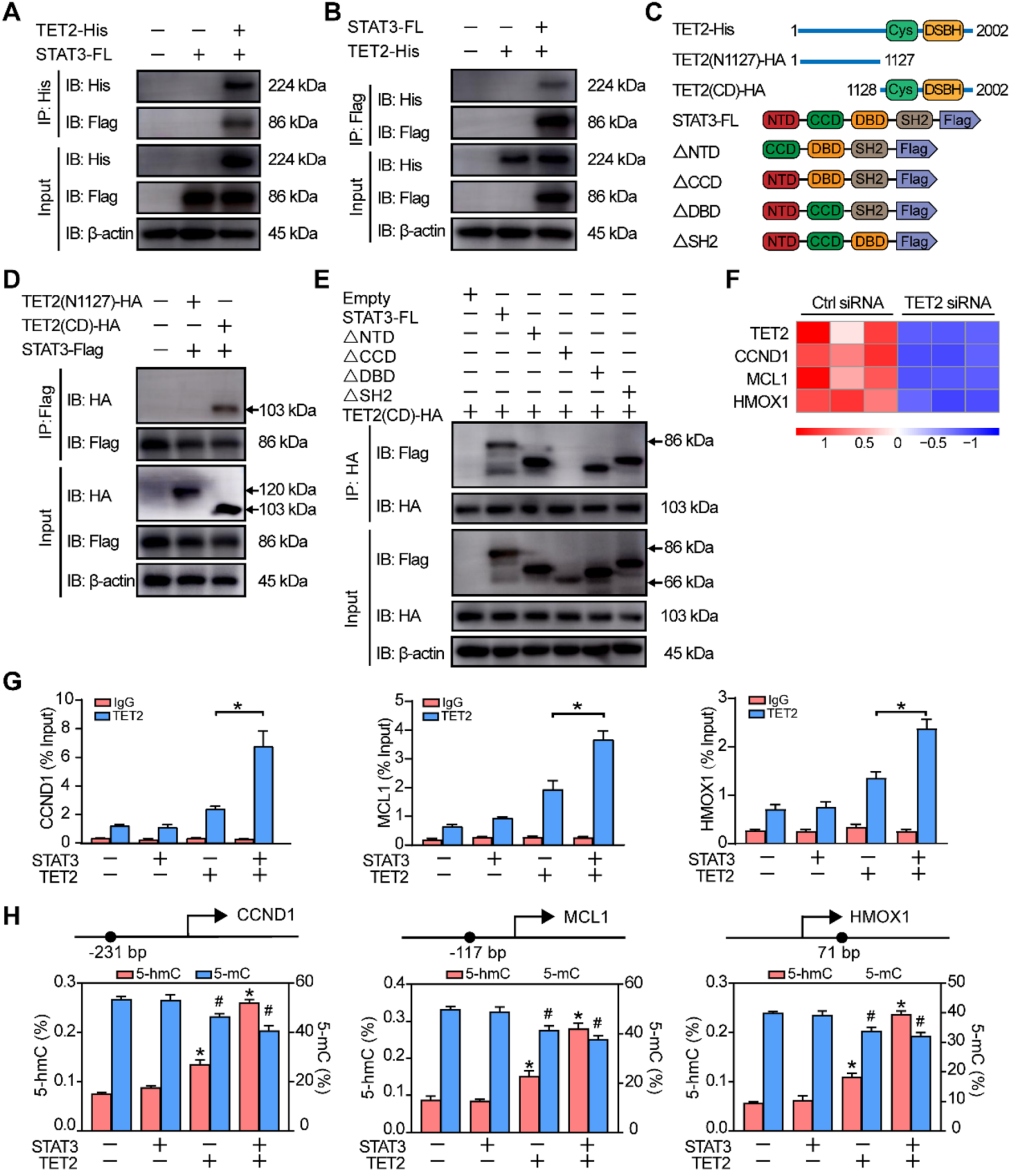
TET2 is recruited by STAT3 and activates its target genes. TET2-His and STAT3-Flag were ectopically expressed in HEK293T and the lysate of HEK293T was immunoprecipitated with **A** anti-His antibody or **B** anti-Flag antibody. **C** Schematic illustration of different plasmids of TET2 and STAT3. Different domains of TET2 and STAT3 were ectopically expressed in HEK293T and the lysate of HEK293T was immunoprecipitated with **D** anti-Flag antibody or **E** anti-HA antibody. **F** Heat map of related genes based on RNA sequencing. **G** The occupancy of TET2 at the promoter regions of STAT3-target genes was determined by ChIP-qPCR. **H** The site-specific 5-hmC and 5-mC levels were determined by using GluMS-qPCR. n = 3 per group. **G**, **H** One-way ANOVA with Bonferroni post hoc test. *, *P* < 0.05 vs. the STAT3 (−) and TET2 (−) group (5-hmC); #, *P* < 0.05 vs. the STAT3 (−) and TET2 (−) group (5-mC)

As a member of TET proteins family, TET2 is different from TET1 and TET3. It lacks a CXXC DNA binding domain and needs a co-factor for binding to DNA, especially transcription factor [33, 34]. To support this hypothesis, co-immunoprecipitation was conducted to determine the binding of TET2 and STAT3. Lysates of HUVECs were immunoprecipitated with anti-TET2 antibodies and anti-STAT3 antibodies respectively. The results suggested that the endogenous TET2-STAT3 association was readily examined in HUVECs (Additional file 1: Fig. S8A, B). To further identify the specificity of TET2-STAT3 interaction, TET2 and STAT3 were ectopically expressed in HEK293T. In endogenous experiments, the interaction of TET2 and STAT3 could be detected in ectopically expressed HEK293T (Fig. 5A, B). To define the binding sites of TET2 and STAT3, a series of plasmids of deletion mutants of both proteins were constructed, containing the CD domain and N-terminal region of TET2, ΔNTD domain, ΔCCD domain, ΔDBD domain and ΔSH2 domain of STAT3 (Fig. 5C). The Co-IP in HEK293T demonstrated that STAT3 bound to the CD domain of TET2, but not its N-terminal region (Fig. 5D). Similar mapping experiments suggested that the CCD domain of STAT3 was the essential region for binding toTET2 (Fig. 5E).

From the analysis of RNA sequencing, we found that CCND1, MCL1 and HMOX1, the target genes of STAT3, were significantly decreased by TET2 knockdown (Fig. 5F), which were correlated with angiogenesis [35–37]. To identify the role of STAT3 in TET2 recruitment, chromatin immunoprecipitation and quantitative PCR (ChIP-qPCR) analysis were conducted to demonstrate the binding of ectopically expressed TET2 to the promoter of STAT3-target genes. Remarkably, the co-expression of TET2 and STAT3 significantly increased the occupancy of TET2 on the promoters of STAT3-target genes (Fig. 5G). Xu et al. [38] reported that glucosylated hydroxymethyl-sensitive qPCR (GluMS-qPCR) could quantify the 5-hmC and 5-mC levels at the specific locus. Therefore, we performed GluMS-qPCR to quantify the levels of 5-hmC and 5-mC at particular nucleotide loci near the transcription start sites of STAT3-target genes. The data revealed that the co-expression of STAT3 and TET2 in HUVECs significantly increased the level of 5-hmC and mildly decreased the level of 5-mC at the promoters of STAT3-target genes (Fig. 5H). To conclude, these findings confirm that STAT3 recruits TET2 to specific genomic regions to control gene expression.

### TET2 promotes angiogenesis in a STAT3-dependent manner

**Fig. 6:**
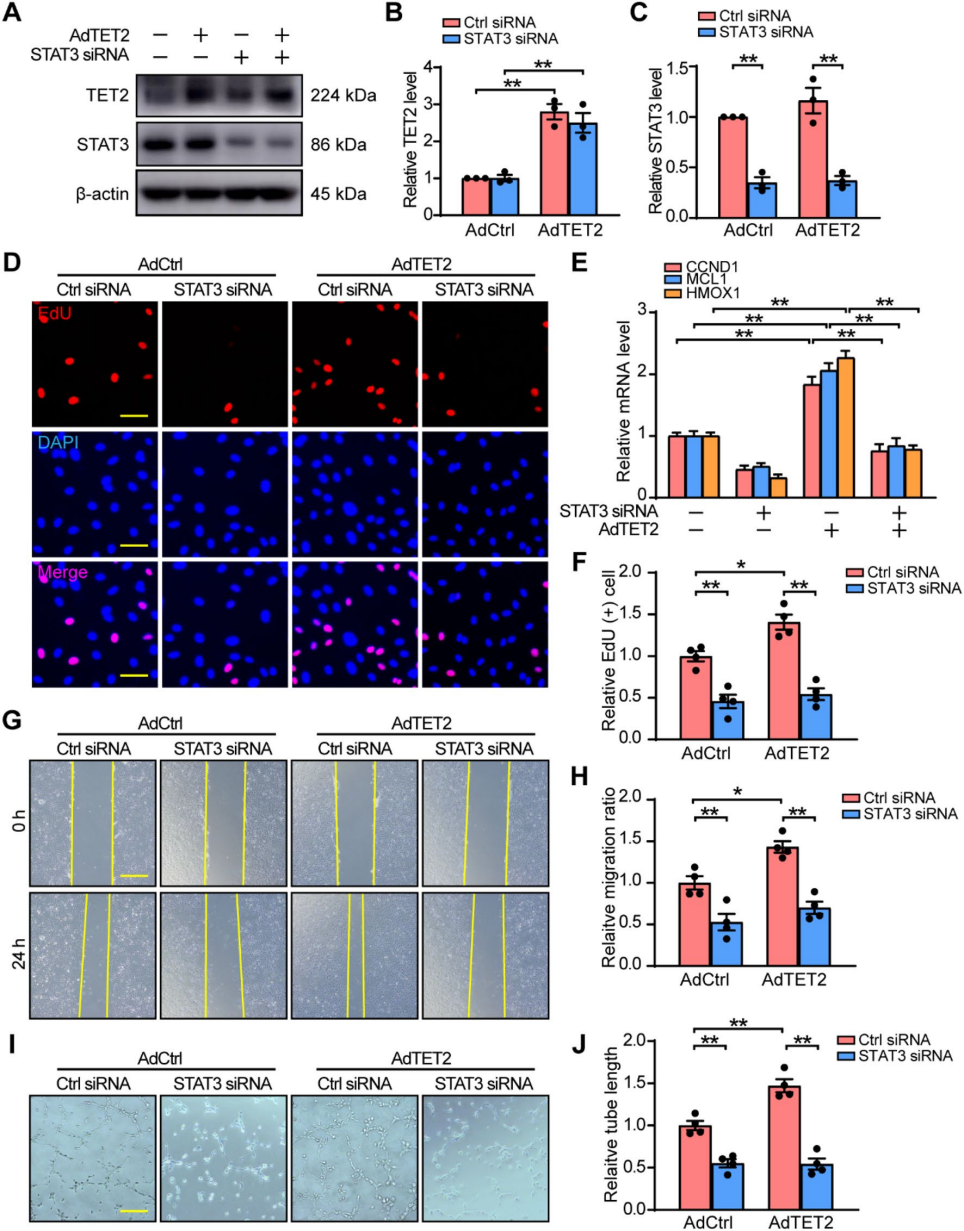
TET2 promotes angiogenesis in a STAT3-dependent manner. **A**, **B**, **C** Western blot of HUVECs infected with TET2 adenovirus in presence or absence of STAT3 siRNA under hypoxia **D, F** EdU assay of HUVECs infected with TET2 adenovirus in presence or absence of STAT3 siRNA under hypoxia. Scale bar, 50 μm. **E** Quantification of the mRNA expression levels of CCND1, MCL1 and HMOX1 in HUVECs infected with TET2 adenovirus in presence or absence of STAT3 siRNA under hypoxia. **G**, **H** Scratch assay and **I**, **J** Tube formation assay of HUVECs infected with TET2 adenovirus in presence or absence of STAT3 siRNA under hypoxia. Scale bar, 100 μm. n = 3–4 per group. **B**, **C**, **F**, **H**, **J** Two-way ANOVA with Bonferroni post hoc test. **E** One-way ANOVA with Bonferroni post hoc test. *, *P* < 0.05; **, *P* < 0.01

To examine the physiological significance of the binding of TET2 and STAT3, we investigated the functional interaction of TET2 and STAT3 in angiogenesis. We first determined the efficiency of TET2 overexpression and STAT3 knockdown in HUVECs by western blot under hypoxia (Fig. 6A–C). Then we investigated how the ectopically expressed TET2 affected the STAT3-target genes in presence or absence of STAT3 under hypoxia. The result of RT-qPCR showed that the effect of TET2-mediated activation of STAT3-target genes was mostly abrogated by STAT3 knockdown (Fig. 6E). Furthermore, the functions of HUVECs were detected with TET2 ectopically expressed in presence or absence of STAT3 siRNA. Compared with the control group, HUVECs with TET2 overexpression displayed better capacities of proliferation (Fig. 6D, F), migration (Fig. 6G, H), and tube formation (Fig. 6I, J) under hypoxia. However, the promotion effects of TET2 on HUVECs were abolished by STAT3 knockdown. Collectively, these results suggest that the effects of TET2 on angiogenesis are in a STAT3-dependent manner.

## Discussion

Angiogenesis is critical to maintaining vessel homeostasis under pathological condition, such as wound healing, and ischemia. In our study, we found the effect of TET2 on regulating angiogenic potential of ECs under pathological conditions and in regulating post‐ischemic angiogenesis, via activating STAT3 target genes. According to the present results, TET2 was a master DNA demethylase that regulated angiogenesis and post‐ischemic, thus providing evidence for the potential therapeutic role of targeting TET2 in the treatment of ischemic diseases. To the best of our current knowledge, our study is the primary research reporting the significant correlation between reduced expression level of 5-hmC and severity of ischemic disease. Remarkably, we illustrated the mechanism of TET2 in angiogenesis in response to ischemic injury, as the deficiency of TET2 aggravates the function of endothelial cells, while the overexpression of TET2 greatly alleviates the dysfunction of endothelial cells in ischemic injury. Consequently, TET2 has the potential to be a promising angiogenic target for clinical application in impaired angiogenesis diseases.

TET proteins, including TET1, TET2 and TET3, were identified as DNA demethylases with critical roles in many physiological processes [25, 39, 40]. TET enzymes are Fe^2+^ and α-ketoglutarate-dependent dioxygenases, which are used by TET enzymes to oxidize 5-mC to 5-hmC. 5-hmC is further oxidized to 5-fC, 5-caC subsequently and the latter two modified bases are excised by TDG to achieve demethylation [18, 20, 41]. Our results showed that 5-hmC was significantly involved in hypoxia-induced dysfunction of endothelial cells, which was mainly regulated by TET2, rather than TET1 or TET3. In response to hypoxia, IL-6, or VEGF, the Janus kinases or intrinsic tyrosine kinases of growth factor receptors phosphorylate STAT3, and lead to the formation of STAT3 dimers, followed by translocation into the nuclear, which is necessary for STAT3’s transcriptional activity [42–44]. RNA sequencing of our study demonstrated that the STAT3 signaling pathway was significantly inhibited by TET2 knockdown. There are many factors affecting the transcriptional activity of STAT3, mainly including directly altering the expression level and the nuclear translocation rate of STAT3. A study in brain tumor cells suggested that downregulation of TET1 led to a significant reduction of STAT3 in transcript level [45]. Considering that TET2 had a similar ability to TET1 on demethylation, we detected the protein level of STAT3 in HUVECs with the knockdown of TET2 and the result showed that STAT3 expression or phosphorylation level were unaffected by TET2. Our previous study showed that NLRC5 could enhance the accumulation of STAT3 in the nucleus to promote STAT3 transcriptional activity, therefore we wondered that the function of TET2 is similar to NLRC5 [32]. Immunoblotting of extraction of cytoplasmic and nuclear proteins, and immunofluorescence both revealed that knockdown of TET2 did not affect the nuclear translocation of STAT3.

Unlike TET1 and TET3, TET2 does not have a CXXC DNA binding domain so that it needs co-factors for binding to DNA [19, 34]. Previous studies reported that TET2 could be recruited by some transcription factors to regulate their downstream genes, such as WT1, Zscan4, and SNIP1 [46–48], which led us to investigate whether TET2 and STAT3 had the same role in endothelial cells. Similarly, our findings indicated the mechanism that TET2 was recruited by STAT3 to a specific sequence in the genome and played the role of DNA demethylase to convert 5-mC to 5-hmC at specific nucleotides in STAT3 target genes. We illustrated the interaction between TET2 and STAT3 from three aspects: First, Co-IP directly indicated the physical binding between TET2 and STAT3. Second, ChIP-qPCR suggested the binding of TET2 to the promoters of STAT3-target genes and the bindings could be regulated by the expression of STAT3. Third, GluMS-qPCR showed that the ability of TET2 converting 5-mC to 5-hmC at the promoter of CCND1, MCL1, HMOX1 was dependent on the expression of STAT3. Meanwhile, the function of TET2 in the endothelial cells was in a STAT3-dependent manner, which was further supported by the knockdown of STAT3 in the endothelial cells with TET2 overexpressed. In concordance with the data of Wang et al. [46], we proved that the CD domain of TET2 was the critical region for playing the role of demethylase. More importantly, it seemed that TET2 could be recruited by different transcription factors in different kinds of cells, as TET2 could bind to SALL4A [49] and EGR2 [50] in other cells. In addition to proving the binding of TET2 and STAT3, we further identified that the CD domain of TET2 and CCD domain of STAT3 were essential for the combination of them by constructing multiple domains of the two proteins. It is necessary to identify the critical domain of TET2 because TET2 is a macromolecular protein, which limited its clinical transformation. As the critical region of TET2, the CD domain might have the same effect as TET2 in promoting endothelial angiogenesis, what is the future direction of our research to reveal the role of the CD domain in angiogenesis.

## Conclusions

In conclusion, our study provides new insights into the role of TET2 in pathological angiogenesis. The deficiency of TET2 in ECs in vitro and in vivo both reveal the inhibitory effects on angiogenesis. Mechanistically, TET2 is recruited by STAT3 to bind with its target genes and increases the 5-hmC level to activate the transcription of the target genes. Collectively, we revealed the potential implication of TET2 in the treatment of ischemic disease.

## Methods

### Cell culture

Human umbilical vein endothelial cells (HUVECs, Cat#8000, ScienCell) were cultured at a 37 ℃ incubator in ECM (Cat#1001, ScienCell) with 5% FBS (Cat#0025, ScienCell), 1% ECGS (Cat#1052, ScienCell) and 1% P/S (Cat#0503, ScienCell). For normoxic treatment, HUEVCs were grown at atmospheric oxygen concentrations (21%) with 5% CO_2_. For hypoxic treatment, they were exposed to an atmosphere of 1% oxygen, 5% CO_2_ and 94% N_2_. Human embryonic kidney 293 T (HEK293T) cells were purchased from Shanghai Zhongqiaoxinzhou Biotechnology Co., Ltd. (China) and cultured in DMEM supplemented with 10% FBS and 1% P/S.

### Small interfering RNAs, plasmid transfection and adenovirus infection

HUVECs or HEK293T were transfected with small interfering RNAs (siRNAs) or plasmid using jetPRIME (Cat#114, Polyplus-transfenction) and infected with adenovirus according to the manufacturer’s protocol. The sequences of siRNAs were listed in Additional file 2: Table S1. The plasmid of STAT3, ΔNTD domain (2–120 aa delated), ΔCCD domain (141–313 aa delated), ΔDBD domain (325–464 aa delated), ΔSH2 domain (584–647 aa delated) and TET2 were constructed by Shanghai Genechem Co., Ltd., as well as the adenoviruses. The plasmid of N-terminal region of TET2 (N1127) and CD domain of TET2 were obtained from Prof. Dan Ye (Fudan University).

### Proliferation assay

EdU Cell Proliferation kit (Cat#C10339, Invitrogen) was used to determine the cell proliferation. After starvation, HUVECs were cultured with EdU-labeling mixture (10 mM) under hypoxia for 12 h. Then cell proliferation was detected according to the manufacturer’s instruction. In summary, 4% paraformaldehyde was used to fix HUVECs, and 0.5% TritonX‐100 was used to permeabilized them. Subsequently, HUVECs were incubated with 1 × Click-iT EdU reaction mixture and stained with Hoechst 33342 for another 15 min. The cell proliferation rate was calculated as EdU-positive cells/total cells of each field.

### Scratch assay

A cross-scratch wound was made by a 200 μl pipette tip in the center of the well after starvation of transfected HUVECs under hypoxia. Pictures were taken from each well after 24 h culture. Wound areas were analyzed by ImageJ software (v1.52a, National Institutes of Health).

### Tube formation assay

Tube formation was detected in a 24-well plate using Matrigel (Cat#354230, Corning), on which transfected HUVECs (2 × 10^5^ cells/well) were seeded under hypoxia. After 8 h culture, pictures were taken from each well. The tube length and tube number were measured by ImageJ software (v1.52a, National Institutes of Health).

### Apoptosis assay

After being transfected with siRNA or infected with adenovirus for 24 h, HUVECs were incubated under hypoxia. After 48 h hypoxia, HUVECs were digested by trypsin and resuspended in binding buffer. 5 μl PI was added into HUVECs and flow cytometry was analyzed with 1 h.

### RNA extraction and RT-qPCR

Total RNAs were isolated using TRIzol Reagent (Cat#15596026, Invitrogen). 1 µg of total RNAs was used for reverse transcription by HiScript III RT SuperMix (Cat#R323, Vazyme) and qPCR was performed using ChamQ Universal SYBR qPCR Master Mix (Cat#711, Vazyme). Quantitative analysis was performed with the 2^−△△CT^ method. The sequence of primers was listed in Additional file 2: Table S2.

### Western blot

Proteins were extracted from cells by Cell Lysis Buffer (Cat#9803 s, Cell Signaling Technologies) and determined using the BCA Protein Quantification Kit (Cat#20201ES76, Yeasen). 30 μg protein for each group was separated on the SDS‐PAGE and transferred to PVDF membranes. After being blocked with 5% BSA at room temperature for 1 h, membranes were incubated with primary antibodies at 4 °C overnight. The next day, they were incubated with the HRP‐linked secondary antibodies at room temperature for 1 h and exposed to ECL substrate (Cat#180–5001, Tanon). The Amersham Imager 600 system (GE Healthcare, USA) was used to develop the membrane and ImageJ software (v1.52a, National Institutes of Health) was used for quantification. The primary and secondary antibodies were listed in Additional file 2: Table S3.

### Co-immunoprecipitation

Cell lysis buffer (Cat#9803, Cell Signaling Technologies) with protease inhibitors (Cat#04693159001, Roche) was used to harvest HUVECs or HEK293T. After determination by the BCA Protein Quantification Kit (Cat#20201ES76, Yeasen), 500 µg of cell lysate was incubated with the primary antibodies at 4 °C overnight. The next day, 20 µl of protein A/G agarose (Cat#sc-2003, Santa Cruz) was used to purify the cell lysate at 4 °C for 4 h. The immunoprecipitated proteins were then used for western blot. The antibodies were listed in Additional file 2: Table S3.

### Extraction of cytoplasmic and nuclear proteins

The cytoplasmic and nuclear proteins were extracted from HUVECs using the kit of NE-PER Nuclear and Cytoplasmic Extraction Reagent (Cat#78,833, Invitrogen). Briefly, HUVECs were transfected with siRNAs for 48 h and then treated with IL-6 (20 ng/mL, Cat#200–06, PeproTech) for 0, 15, 30, 60 min. After digestion by trypsin, HUVECs were suspended in completed medium. The portions of cytoplasm and nucleus were isolated extracted according to the instruction of manufacturer.

### DNA extraction

TIANamp Genomic DNA Kit (Cat#DP304, Tiangen) was used to isolate genomic DNAs from cultured cells. Briefly, cultured cells were treated with lysis buffer and released genomic DNAs. Next, RNaseA and proteinase K were used to remove RNAs and histone. Then the spin column bound with DNAs and centrifugation processing removed contaminants and enzyme inhibitors. Purified DNAs were eluted in low-salt buffer and ready for use in downstream applications.

### Dot blot

Total DNAs were extracted as previously mentioned and diluted to 100 ng/μl. 2 μl of total DNAs were dropped onto a Hybond‐N^+^ membranes and cross‐linked by UV for 30 s. Then, the membranes were blocked with 5% BSA at room temperature for 1 h and incubated with anti-5-hmC antibody at 4 °C overnight. After being washed three times, they were incubated with HRP‐linked secondary antibody at room temperature for 1 h and exposed to ECL substrate (Cat#180–5001, Tanon). The Amersham Imager 600 system (GE Healthcare, USA) was used to develop the membrane and ImageJ software (v1.52a, National Institutes of Health) was used for quantification. The antibodies were listed in Additional file 2: Table S3.

### ELISA

Genomic DNA methylation was measured by the Global DNA Methylation-LINE-1 Kit (Cat#55017, Active Motif). Briefly, the genomic DNAs were enzymatically digested using MseI enzyme to generate the appropriate fragments to hybridize to a biotinylated consensus sequence corresponding to human LINE-1 transposon. Hybridized samples were immobilized to a streptavidin-coated 96-well plate while unbound DNA fragments were washed away. Methylated cytosines were identified using a 5-methylcytosine antibody, HRP-conjugated secondary antibody and colorimetric detection reagents. The colorimetric readout was quantified by microplate reader at 450 nm. Generating a standard curve using the included DNA standards with known LINE-1 methylation levels provides the relative level of 5-methylcytosine in each DNA sample.

### ChIP-qPCR

Chromatin immunoprecipitation (ChIP) assays were performed using a SimpleChIP Enzymatic Chromatin IP kit (Cat#9003, Cell Signaling Technologies). HUVECs were crosslinked with 1% paraformaldehyde and the chromatin was fragmented with nuclease and sonication. Then the fragmented chromatin was incubated with anti-TET2 antibody or negative control IgG overnight. The next day, protein G magnetic beads were used to incubate with chromatin. After being washed and purified, precipitated DNAs were analyzed by qPCR. Primers used for ChIP-qPCR are listed in Additional file 2: Table S2.

### GluMS-qPCR

The 5-hmC and 5-mC levels in TET2-binding regions were detected by EpiMark 5-hmC and 5-mC Analysis Kit (Cat# E3317S, New England Biolabs). In short, genomic DNAs were extracted as previously mentioned and treated with T4 β- glucosyltransferase, by which all 5-hmC would be glucosylated to 5-ghmC. Then the modified DNAs were cleaved by restriction endonucleases, including MspI and HpaII. MspI would cleave 5-mC and 5-hmC, but not 5-ghmC, while HpaII would cleave only a completely unmodified site: any modification (5-mC, 5-hmC or 5-ghmC) at either cytosine blocked cleavage. The MspI- and HpaII-resistant fractions were quantified by qPCR and normalized to the mock digestion. Primers used for GluMS-qPCR were listed in Additional file 2: Table S2.

### RNA sequencing

HUVECs were transfected with control siRNA (Ctrl siRNA) or TET2 siRNA (50 nM) for 24 h, followed by 48 h of hypoxia. RNA sequencing was conducted by Berry Genomics (China). In brief, Trizol reagent was used to harvest total RNAs and 1 µg of total RNAs was used for constructing RNA libraries by Illumina TruSeq RNA Sample Prep Kit (Cat#FC-122–1001, Illumina). RNA libraries for sequencing were prepared using the standard Illumina protocols, and RNA sequencing was performed by the Illumina NovaSeq6000 platform. DESeq (version 1.22.1) was used to screen the differentially expressed genes. The raw data of RNA sequencing were deposited in the Gene Expression Omnibus (Accession Number: GSE200080).

### GVSA

To explore the underlying changes in pathway activity between two groups with significant differences, we performed gene set variation analysis (GSVA) to calculate the scores for each group based on HALLMARK gene sets. Enriched gene sets were assigned based on control *P*-value < 0.05 as significantly altered.

### GSEA

Gene set enrichment analysis (GSEA, version 4.1.0) was used to determine whether a genetically defined genome is statistically significant between HUVECs transfected with control siRNA and TET2 siRNA under hypoxia. We constructed RNK file based on genes’ log2FoldChange and − log10P.value. GSEA was performed with default algorithm as 1000 permutations, minimum term size of 15, and maximum term size of 500. We served HALLMARK as our annotated gene sets, which were collected from the Molecular Signature Database 3.0. Enriched gene sets were assigned based on normalized *P*-value < 0.05 and FDR q-value < 0.25.

### Animals

The wild-type mice (WT, C57BL/6) were purchased from Vital River Laboratory. CDH5Cre mice (B6-Cdh5^tm1(iCre/ERT2)^/Bcgen) were purchased from Biocytogen Co., Ltd. TET2-floxed mice (B6;129S-Tet2^tm1.1Iaai^/J) and mTmG mice (B6.129(Cg)-Gt (ROSA)26Sor^tm4(ACTB−tdTomato,−EGFP)Luo^/J) were purchased from The Jackson Laboratory. The primers used for genotyping were listed in Additional file 2: Table S2. After continuous cross breeding, TET2^flox/flox^ mice, CDH5Cre; TET2^flox/flox^ mice, CDH5Cre; mTmG mice and CDH5Cre; TET2^flox/flox^; mTmG mice were generated. To obtain TET2^EC−KO^ mice and TET2^EC−KO^; mTmG mice, 8-week-old male CDH5Cre; TET2^flox/flox^ mice and CDH5Cre; TET2^flox/flox^; mTmG mice were injected with tamoxifen (75 mg/kg body weight, Cat#T5648, Sigma) for 5 consecutive days. The control mice received the same treatment. Animal studies were conducted in compliance with the Guide for the Care and Use of Laboratory Animals published by the NIH and approved by the Animal Care and Use Committees of Shanghai Tenth People’s Hospital.

### Hindlimb ischemia model

The hindlimb ischemia (HLI) model was performed as previously described [26]. After anesthetization with 2% isoflurane, the hair of hindlimb was removed. The left femoral artery was ligated and excised, while sham operations were performed on the contralateral hindlimb. Perfusion recovery was detected by PeriCam PSI System (PeriMed, Sweden) on day 0, 3, 7, 14 and 21 post-HLI. The ischemic limb perfusion was normalized to the sham limb for each mouse.

### Tumor transplant

8 week-old male TET2^flox/flox^ and TET2^EC−KO^ mice underwent xenograft experiments. The B16F10 mouse melanoma cells were provided by Prof. Ping Wang of Tongji University. Cells were cultured in DMEM complete medium (high glucose, 5% FBS, 0.5% PS) and suspended in PBS (1 × 10^7^ cells/mL), then injected subcutaneously in the murine flank (100 µl/mouse). The volume of tumors was calculated as 4π/3 × (width/2)^2^ × (length/2) by measuring the length and width every 2 days. Then mice were euthanized and tumors were dissected 12 days after injection.

### Immunofluorescence and histology

Tissues of mice were harvested and sliced into 5 µm-thick Sect.  4% formaldehyde-fixed sections were incubated in 10% normal goat serum and 0.5% Triton-X 100 for half an hour. DAPI (1:5000, Vector Laboratories) was used to stain the nuclei for 30 min. All steps should be operated in the dark. Olympus IX83 fluorescence microscope (Olympus Corporation, Japan) was used to obtain images. H&E staining was used to quantify tissue necrosis and regenerating muscle fibers, and Masson staining was used to quantify tissue fibrosis. Necrotic tissues were identified by morphological alterations of myofibers or loss of sarcolemmal integrity, the presence of cellular debris and many mononuclear cell infiltrates in the surrounding interstitial space. Regenerating tissues were identified by the predominant presence of fibers with centrally positioned nuclei and/or some lingering mononuclear cell infiltrates. The quantification was performed by ImageJ software (v1.52a, National Institutes of Health), and expressed as the ratio of positive area to the total surface area of the tissue section.

### Flow cytometry analysis

The flow cytometry analysis was performed as Liu et al. [27] described. Briefly, the muscles of anesthetized mice were cut into small pieces and digested in a mixture of 5 mL HBSS containing 1 mL collagenase II (100 U/mL, Cat#1148089, Sigma-Aldrich) and 2 mL (1 U/mL) dispase (Cat#354235, Corning) at 37 °C for 1 h. Next, the tissues were digested in 1 U/mL dispase for 30 min. After digestion, the cells were filtered with 70 μm filters and red blood cells were lysed in FACS Lysing Solution (Cat#349202, BD Pharmingen) for 5 min at room temperature. Finally, cells were resuspended in 0.1% BSA solution.

### Statistical analysis

Each study was performed with at least 3 independent experiments and run-in triplicate. Data were calculated and presented as the mean ± SEM. For two groups, statistical significance was determined by Student’s *t*-test if data had equal variance and by Mann–Whitney test if data had unequal variance. For more than two groups, statistical significance was determined by one-way or two-way ANOVA with Bonferroni post hoc test. *P*-value < 0.05 was defined as statistical significance. The statistical analysis was performed using GraphPad Prism (v6.01, GraphPad Software).

## Supplementary Information

**Additional file 1: Figure S1.** The HIF signaling pathway under hypoxia. **Figure S2.** Gating strategy used in the flow cytometry of the apoptosis assay. **Figure S3.** TET2 overexpression alleviates the hypoxia‐induced dysfunction of HUVECs. **Figure S4.** Creation of TET2^EC-KO^; mTmG mice. **Figure S5.** Deficiency of TET2 in endothelial cells impairs tumor growth. **Figure S6.** TET2 knockdown did not affect the expression STAT3. **Figure S7.** TET2 knockdown has no effect on the translocation of STAT3. **Figure S8.** Endogenous binding of TET2 and STAT3 in HUVECs.

**Additional file2: Table S1.** The list of siRNA sequence. **Table S2.** The list of primer sequence. **Table S3.** The list of antibodies.

## Data Availability

The datasets generated during and/or analyzed during the current study are available from the corresponding author on reasonable request. The raw data of RNA sequencing were deposited in the Gene Expression Omnibus (Accession Number: GSE200080).

## Abbreviations

TET2: Ten–eleven translocation protein
STAT3: Signal transducer and activator of transcription 3
5-mC: 5-methylcytosine
5-hmC: 5-hydroxymethylcytosine
ECs: Endothelial cells
HUVEC: Human umbilical vein endothelial cells
CCND1: Cyclin D1
MCL1: Myeloid cell leukemia 1
HMOX1: Heme oxygenase 1
