# Supplementary material for: Loss of TET2 impairs endothelial angiogenesis via downregulating STAT3 target genes
Source: Cell Biosci. 2023 Jan 19;13:12. doi: 10.1186/s13578-023-00960-5 (PMC9850815; doi:10.1186/s13578-023-00960-5)
Supplement: Supplementary file 1 — Additional file 1: Figure S1. The HIF signaling pathway under hypoxia. Figure S2. Gating strategy used in the flow cytometry of the apoptosis assay. Figure S3. TET2 overexpression alleviates the hypoxia‐induced dysfunction of HUVECs. Figure S4. Creation of TET2EC-KO; mTmG mice. Figure S5. Deficiency of TET2 in endothelial cells impairs tumor growth. Figure S6. TET2 knockdown did not affect the expression STAT3. Figure S7. TET2 knockdown has no effect on the translocation of STAT3. Figure S8. Endogenous binding of TET2 and STAT3 in HUVECs. [file 13578_2023_960_MOESM1_ESM.docx]

**Additional file 1**

**Loss of TET2** **Impairs Endothelial Angiogenesis via Downregulating STAT3 Target Genes**

Yefei Shi^1*^, Bo Li^1*^, Xinru Huang^2*^, Wenxin Kou^1^, Ming Zhai^1^, Yanxi Zeng^1^, Shuangjie You^1^, Qing Yu^1^, Yifan Zhao^1^, Jianhui Zhuang^1^, Wenhui Peng^1#^, and Weixia Jian^2#^

1. Department of Cardiology, Shanghai Tenth People’s Hospital, Tongji University School of Medicine, Shanghai, China

2. Department of Endocrinology, Xinhua Hospital, Shanghai Jiaotong University School of Medicine, Shanghai, China

* Co-first author

# Corresponding authors: Weixia Jian, E-mail: jianweixia@xinhuamed.com.cn. Department of Endocrinology, Xinhua Hospital, Shanghai Jiaotong University School of Medicine, 1665 Kongjiang Road, Shanghai 200092, China; Wenhui Peng, E-mail: pwenhui@tongji.edu.cn, Department of Cardiology, Shanghai Tenth People’s Hospital, Tongji University School of Medicine, 301 Middle Yanchang Road, Shanghai, 200072, China.

**Supplementary Figures**


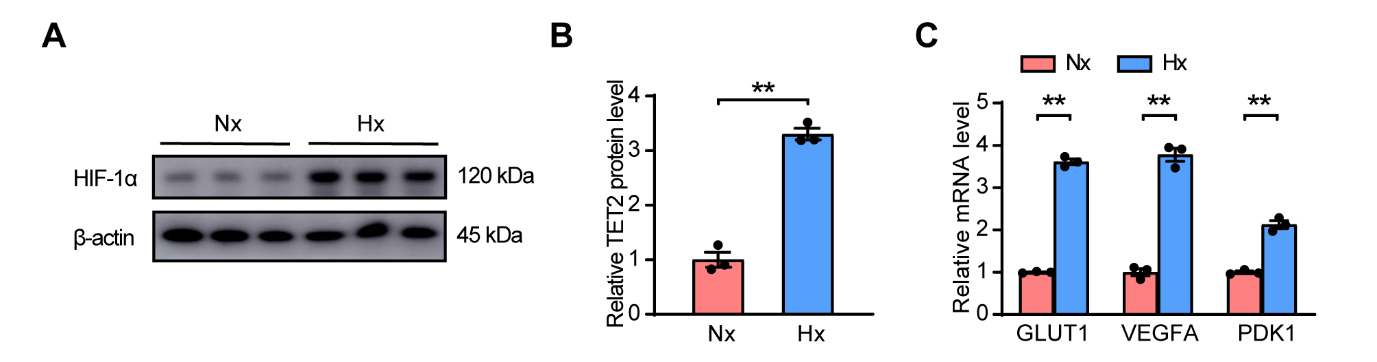


**Fig. S1 The HIF signaling pathway under hypoxia. (A, B)** Western blot of HUVECs under normoxia and hypoxia. **(C)** Quantification of the mRNA expression levels of GLUT1, VEGFA and PDK1 under normoxia and hypoxia. n = 3 per group. **(B, C)** Unpaired Student's *t*‐test. **, *P* < 0.01.


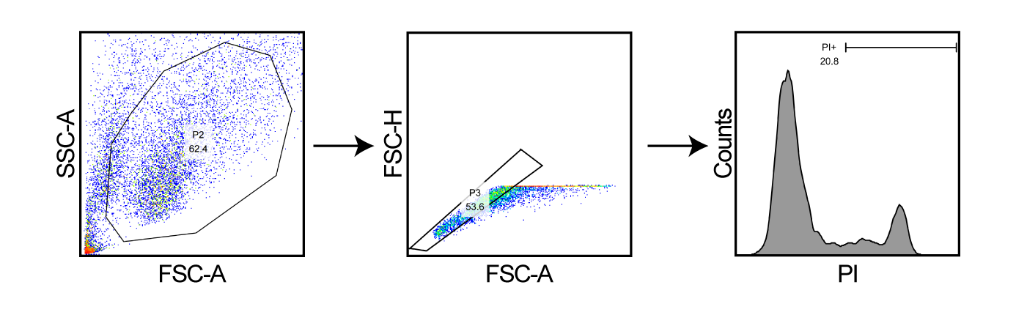


**Fig. S2 Gating strategy used in the flow cytometry of the apoptosis assay.**


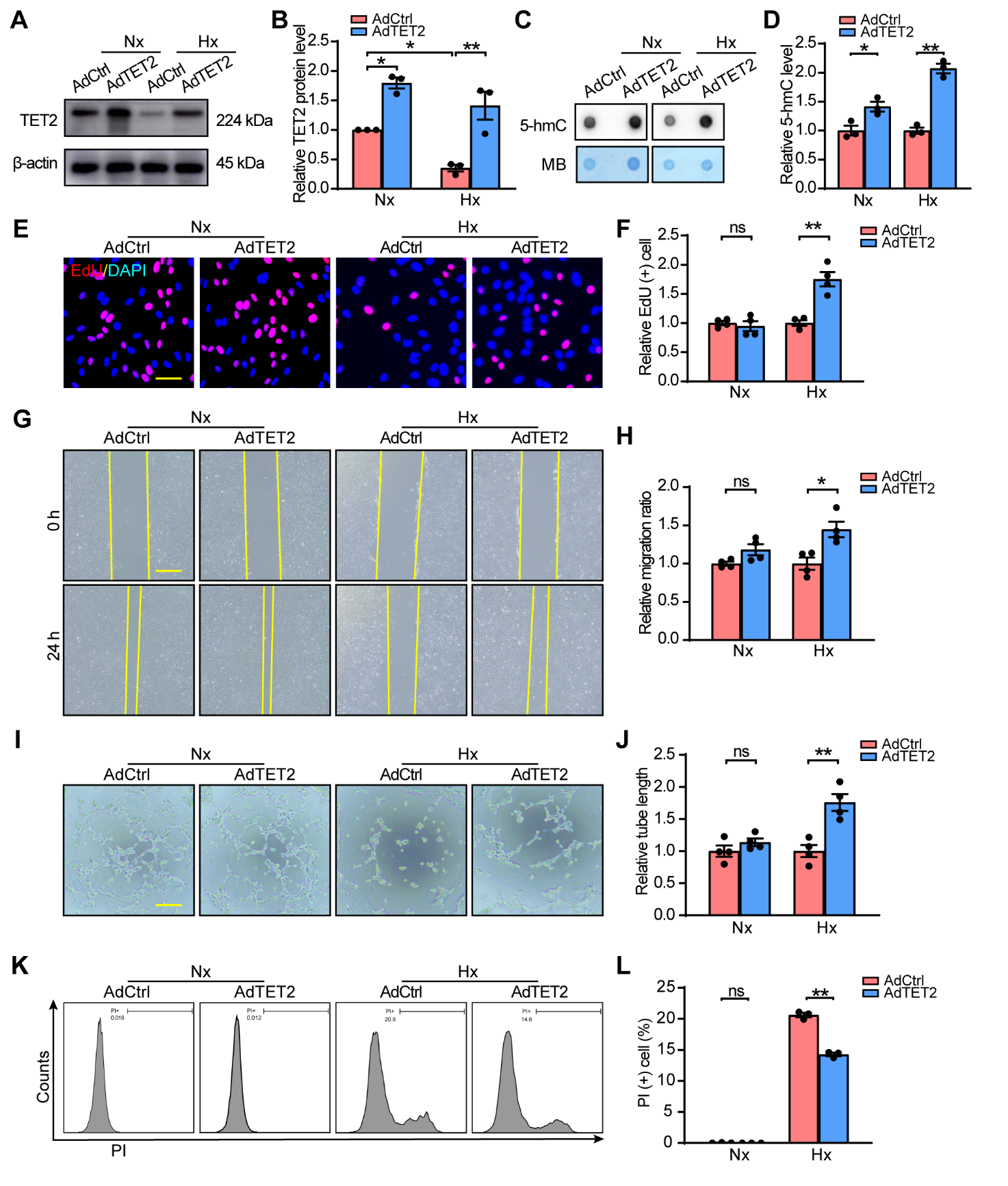


**Fig. S3 TET2 overexpression alleviates the hypoxia‐induced dysfunction of HUVECs. (A, B)** Western blot of HUVECs infected with control adenovirus (AdCtrl) or TET2 adenovirus (AdTET2) under normoxia or hypoxia. **(C, D)** Dot blot assay of global 5-hmC using genomic DNA from HUVECs infected with AdCtrl or AdTET2 under normoxia or hypoxia. **(E, F)** EdU assay, **(G, H)** Scratch assay, and **(I, J)** Tube formation assay of HUVECs infected with AdCtrl or AdTET2 under normoxia or hypoxia. Scale bar, 100 μm. **(K, L)** Apoptosis assay images of HUVECs infected with AdCtrl or AdTET2 under normoxia or hypoxia. n = 3-4 per group. **(B)** Two-way ANOVA with Bonferroni post hoc test. (**D, F, H, J, L**) Unpaired Student's *t*‐test. ns, no significant; *, *P* < 0.05; **, *P* < 0.01.


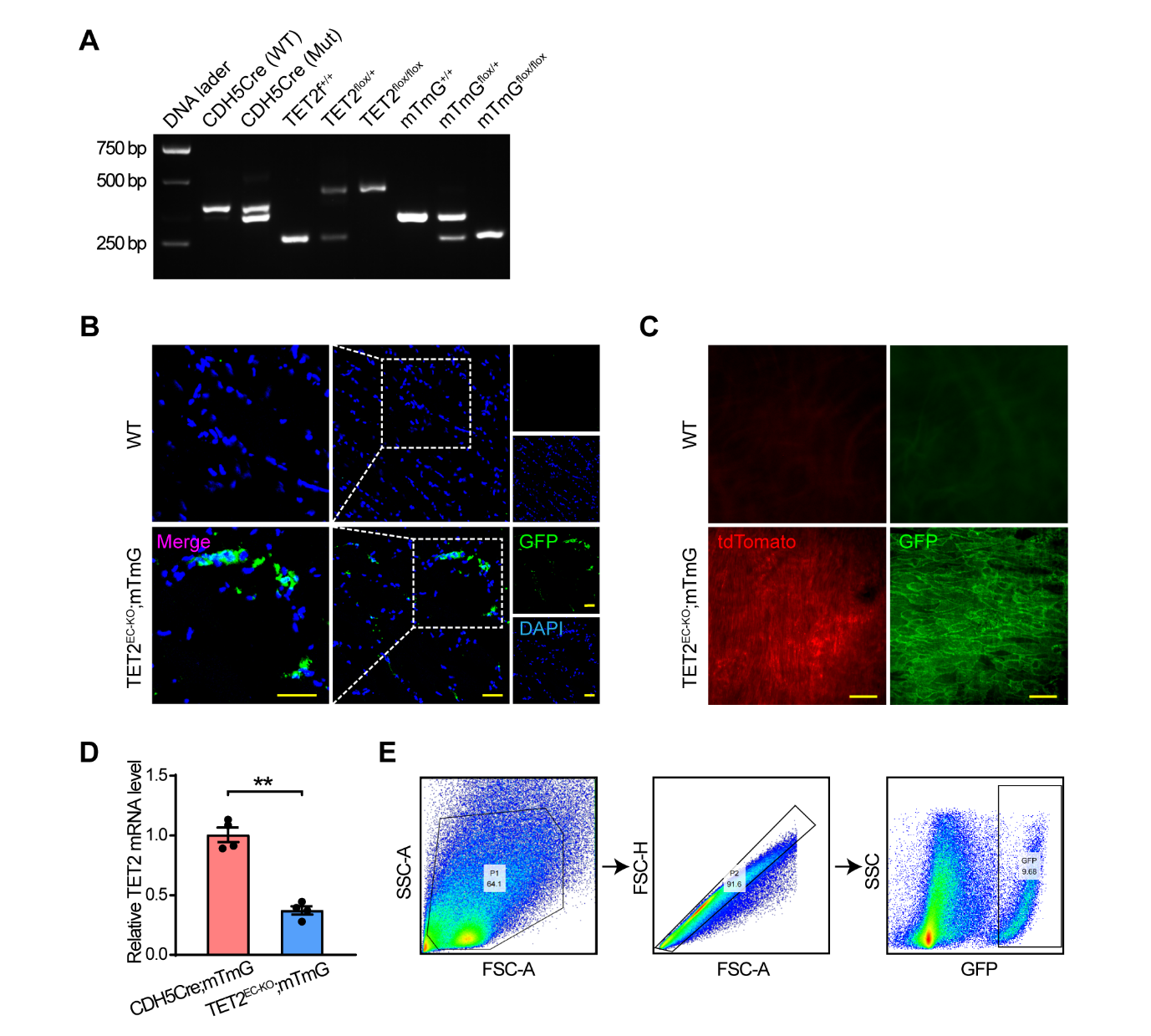


**Fig. S4 Creation of TET2^EC-KO^; mTmG mice.** **(A)** PCR of tail genomic DNA. **(B)** Immunofluorescent staining of GFP-positive cells in the cross section of gastrocnemius muscles from TET2^EC-KO^; mTmG mice. GFP (green), DAPI (blue). **(C)** Immunofluorescent staining of GFP-positive cells in the cross section of aorta from TET2^EC-KO^; mTmG mice. tdTomato (red), GFP (green). **(D)** Quantification of the mRNA expression levels of TET2 in the GFP positive cells separated by flow cytometry from the gastrocnemius muscles of CDH5Cre; mTmG and TET2^EC-KO^; mTmG mice. **(E)** Gating strategy used in the flow cytometry of GFP positive cells. n = 4 per group. (**D**) Unpaired Student's *t*‐test. **, *P* < 0.01.


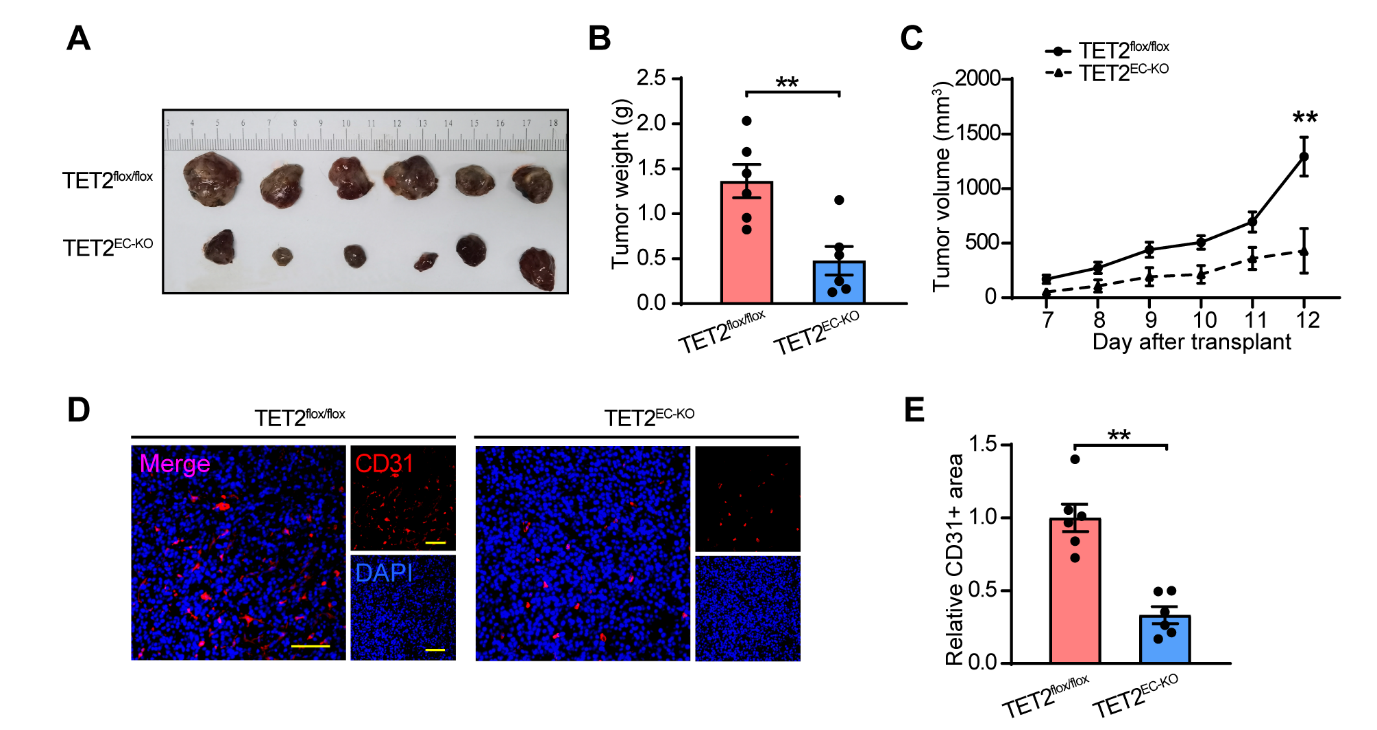


**Fig. S5 Deficiency of TET2 in endothelial cells impairs tumor growth. (A)** The tumor size of TET2^flox/flox^ and TET2^EC-KO^ mice (12 days). **(B)** The tumor weight of TET2^flox/flox^ and TET2^EC-KO^ mice (12 days). **(C)** The volume curves of TET2^flox/flox^ and TET2^EC-KO^ mice after subcutaneous injection of B16F10 cells. **(D, E)** Immunofluorescent staining of the CD31 positive cells quantified in tumors. CD31 (red), DAPI (blue). Scar bar, 100 µm. n = 6 mice per group. (**B, C, E**) Unpaired Student's *t*‐test. **, *P* < 0.01*.*


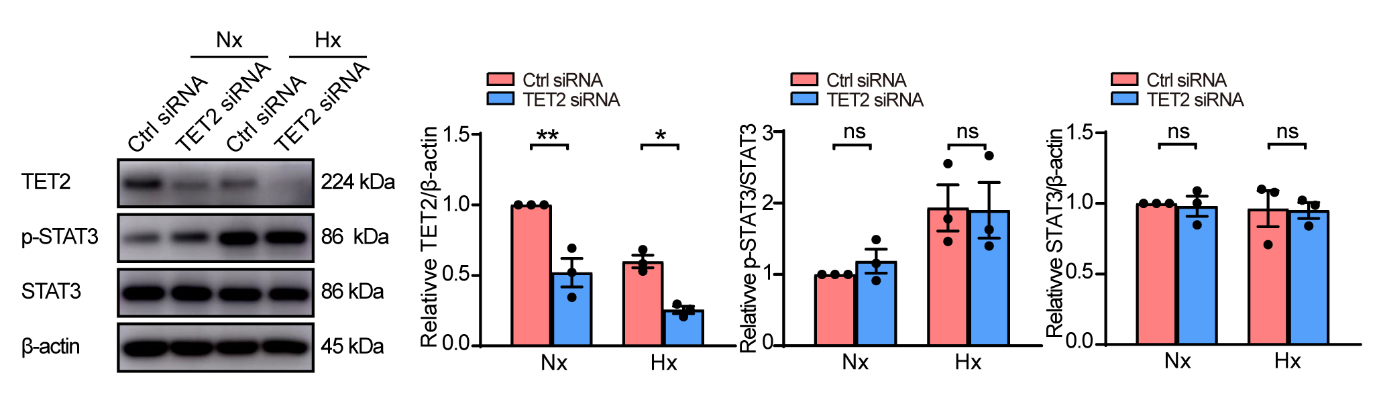
 **Fig. S6 TET2 knockdown did not affect the expression STAT3.** Western blot images of HUVECs transfected with Ctrl siRNA or TET2 siRNA under normoxia or hypoxia. n = 3 per group. Two-way ANOVA with Bonferroni post hoc test. ns, no significant; *, *P* < 0.05; **, *P* < 0.01; Nx, normoxia; Hx, hypoxia.


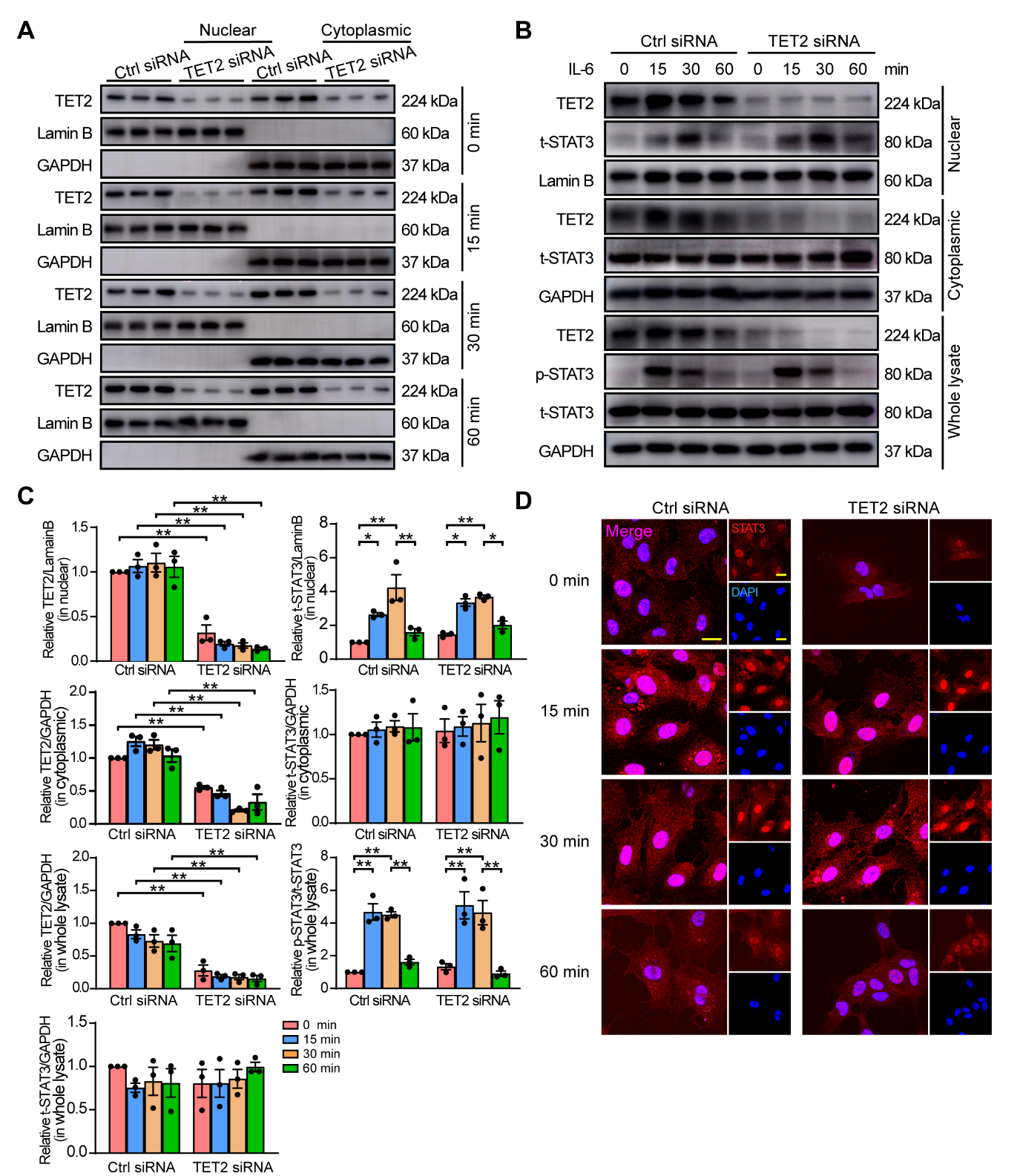


**Fig. S7 TET2 knockdown has no effect on the translocation of STAT3.** **(A, B, C)** The protein levels of TET2, t-STAT3, p-STAT3^Y705^, Lamin B, and GAPDH were measured by western blot in cytoplasmic, nuclear fractions, and whole-cell lysates. **(D)** Confocal microscopy images of immunofluorescence staining for STAT3 and DAPI. STAT3 (red), DAPI (blue). Scale bar, 20 μm. n = 3 per group. (**C**) Two-way ANOVA with Bonferroni post hoc test. *, *P* < 0.05; **, *P* < 0.01.


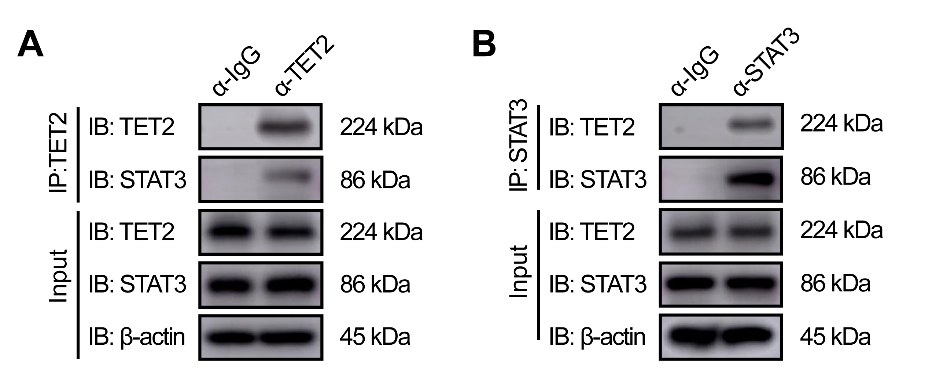


**Fig. S8 Endogenous binding of TET2 and STAT3 in HUVECs.** The lysate of HUVECs was immunoprecipitated with anti-TET2 antibody **(A)** or anti-STAT3 antibody **(B)** and then immunoblotted with the indicated antibodies.
