# Supplementary material for: Loss of TET2 impairs endothelial angiogenesis via downregulating STAT3 target genes
Source: Cell Biosci. 2023 Jan 19;13:12. doi: 10.1186/s13578-023-00960-5 (PMC9850815; doi:10.1186/s13578-023-00960-5)
Supplement: Supplementary file 2 — Additional file2: Table S1. The list of siRNA sequence. Table S2. The list of primer sequence. Table S3. The list of antibodies. [file 13578_2023_960_MOESM2_ESM.docx]

**Additional file 2**

**Loss of TET2** **Impairs Endothelial Angiogenesis via Downregulating STAT3 Target Genes**

Yefei Shi^1*^, Bo Li^1*^, Xinru Huang^2*^, Wenxin Kou^1^, Ming Zhai^1^, Yanxi Zeng^1^, Shuangjie You^1^, Qing Yu^1^, Yifan Zhao^1^, Jianhui Zhuang^1^, Wenhui Peng^1#^, and Weixia Jian^2#^

1. Department of Cardiology, Shanghai Tenth People’s Hospital, Tongji University School of Medicine, Shanghai, China

2. Department of Endocrinology, Xinhua Hospital, Shanghai Jiaotong University School of Medicine, Shanghai, China

* Co-first author

# Corresponding authors: Weixia Jian, E-mail: jianweixia@xinhuamed.com.cn. Department of Endocrinology, Xinhua Hospital, Shanghai Jiaotong University School of Medicine, 1665 Kongjiang Road, Shanghai 200092, China; Wenhui Peng, E-mail: pwenhui@tongji.edu.cn, Department of Cardiology, Shanghai Tenth People’s Hospital, Tongji University School of Medicine, 301 Middle Yanchang Road, Shanghai, 200072, China.

**Supplementary Tables**

**Table S1. The list of siRNA sequence**

| siRNA | Sequence |  |
| --- | --- | --- |
| Ctrl siRNA | ACGUGACACGUUCGGAGAA | |
| TET2 siRNA | AAAGGAACAGGUAUUUAGC | |
| STAT3 siRNA | AGAAUGUUAAAUUUCCGGG | |

**Table S2. The list of primer sequence**

| Gene | Sequence | Application |
| --- | --- | --- |
| TET2 | Forward: AAGAATTGCTACAGGCCTGC | Genotyping |
|  | Reverse: TTCTTTAGCCCTTGCTGAGC |  |
| CDH5 (WT) | Forward: GCTTGCCTTCCCTACTTAGACTAGC | Genotyping |
|  | Reverse: TAGTGGGGCAGCGATTCATTTTTCT |  |
| CDH5 (Mut) | Forward: GCTTGCCTTCCCTACTTAGACTAGC | Genotyping |
|  | Reverse: GCACACAGACAGGAGCATCTTC |  |
| mTmG (WT) | Forward: CTCTGCTGCCTCCTGGCTTCT | Genotyping |
|  | Reverse: CGAGGCGGATCACAAGCAATA |  |
| mTmG (Mut) | Forward: CTCTGCTGCCTCCTGGCTTCT | Genotyping |
|  | Reverse: TCAATGGGCGGGGGTCGTT |  |
| TET1 | Forward: CATCAGTCAAGACTTTAAGCCCT | RT-qPCR |
|  | Reverse: CGGGTGGTTTAGGTTCTGTTT |  |
| TET2 | Forward: TTCGCAGAAGCAGCAGTGAAGAG | RT-qPCR |
|  | Reverse: AGCCAGAGACAGCGGGATTCCTT |  |
| TET3 | Forward: TACCAACCGCCGCACGCAC | RT-qPCR |
|  | Reverse: AGCCGCTCCTTGTCCCCAC |  |
| GLUT1 | Forward: ATTGGCTCCGGTATCGTCAAC | RT-qPCR |
|  | Reverse: GCTCAGATAGGACATCCAGGGTA |  |
| VEGFA | Forward: AGGGCAGAATCATCACGAAGT | RT-qPCR |
|  | Reverse: AGGGTCTCGATTGGATGGCA |  |
| PDK1 | Forward: CTGTGATACGGATCAGAAACCG | RT-qPCR |
|  | Reverse: TCCACCAAACAATAAAGAGTGCT |  |
| β-actin | Forward: CATGTACGTTGCTATCCAGGC | RT-qPCR |
|  | Reverse: CTCCTTAATGTCACGCACGAT |  |
| CCND1 | Forward: GCTGCGAAGTGGAAACCATC | RT-qPCR |
|  | Reverse: CCTCCTTCTGCACACATTTGAA |  |
| MCL | Forward: TGCTGGAGTTGGTCGGGGAA | RT-qPCR |
|  | Reverse: TCGTAAGGTCTCCAGCGCCT |  |
| HMOX1 | Forward: AAGACTGCGTTCCTGCTCAAC | RT-qPCR |
|  | Reverse: AAAGCCCTACAGCAACTGTCG |  |
| CCND1 | Forward: AGATTCTTTGGCCGTCTGTC | ChIP-qPCR |
|  | Reverse: GGGAGACCACGAGAAGGG |  |
| MCL1 | Forward: AGCTGGTAGGTGCCGTGC | ChIP-qPCR |
|  | Reverse: AGTGGCGAGCAGCTCCTTTA |  |
| HMOX1 | Forward: GGTTGCTAAGTTCCTGATGTTG | ChIP-qPCR |
|  | Reverse: CCCTTCTGCTCCCTGTCC |  |
| CCND1 | Forward: GACCCACTCGAGGCGGACG | GluMS-qPCR |
|  | Reverse: CTGCCCTGCGGCGGAGTT |  |
| MCL1 | Forward: GCTGGTAGGTGCCGTGCG | GluMS-qPCR |
|  | Reverse: AGAAGTGGCGAGCAGCTCCT |  |
| HMOX1 | Forward: CGGAGCCAGCACGAACGA | GluMS-qPCR |
|  | Reverse: GCAAGCAGGGTTGGGAGAGA |  |

**Table S3. The list of antibodies**

| Antibody | Catalogue | Dilution | Application |
| --- | --- | --- | --- |
| HRP conjugated Goat Anti-Rabbit IgG (H+L) | Cat#115-036-003,  Jackson Immuno Research | 1:5000 | WB |
| HRP conjugated Goat Anti-Rat IgG  (H+L) | Cat# 112-036-003,  Jackson Immuno Research | 1:5000 | WB |
| HRP conjugated Goat Anti-Mouse IgG (H+L) | Cat# 112-036-003,  Jackson Immuno Research | 1:5000 | WB |
| Donkey anti-Mouse  IgG (H+L), Alexa  Fluor Plus 594 | Cat#32744, Invitrogen | 1:100 | IF |
| Donkey anti-Rat  IgG (H+L), Alexa  Fluor Plus 594 | Cat#21209, Invitrogen | 1:100 | IF |
| TET2 | Cat#ab94580, Abcam | 1:1000 | WB |
| TET2 | Cat#18950, CST | 1:50  1:50 | IP  ChIP |
| STAT3 | Cat#9139, CST | 1:1000  1:50  1:100 | WB  IP  IF |
| p-STAT3 | Cat#9145, CST | 1:1000 | WB |
| HIF-1α | Cat#36169, CST | 1:1000 | WB |
| β-actin | Cat#3700, CST | 1:1000 | WB |
| Lamin B | Cat# BA1228, Boster | 1:1000 | WB |
| GAPDH | Cat#60004-1-Ig, Proteintech | 1:5000 | WB |
| His | Cat#12698, CST | 1:1000  1:50 | WB  IP |
| Flag | Cat#8146, CST | 1:1000  1:50 | WB  IP |
| HA | Cat#3724, CST | 1:1000  1:50 | WB  IP |
| CD31 | Cat#550274, BD Biosciences | 1:100 | IF |
| 5-hmC | Cat#39770, Active Motif | 1:5000 | Dot blot |
